# External and internal influences yield similar memory effects: the role of deception and suggestion

**DOI:** 10.3389/fpsyg.2023.1081528

**Published:** 2023-08-28

**Authors:** Henry Otgaar, Ivan Mangiulli, Fabiana Battista, Mark L. Howe

**Affiliations:** ^1^Leuven Institute of Criminology (LINC), Catholic University of Leuven, Leuven, Belgium; ^2^Faculty of Psychology and Neuroscience, Maastricht University, Maastricht, Netherlands; ^3^Department of Education, Psychology, and Communication Sciences, University of Bari Aldo Moro, Bari, Italy; ^4^Department of Psychology, City, University of London, London, United Kingdom

**Keywords:** lying, suggestion, forgetting, false memory, cognitive dissonance

## Abstract

In legal cases, testimonies can become contaminated because of an amalgam of external and internal influences on memory. It is well-established that external influences (e.g., suggestive interviews) can hurt memory. However, less focus has been placed on the impact of internal influences (e.g., lying) on memory. In the current review, we show that the available evidence suggests that both external and internal influences exert similar effects on memory. That is, we review studies showing that suggesting non-occurrences and suggesting non-experiences can lead to omission errors and false memories, respectively. Likewise, these memory effects are also observed when focusing on internal influences. That is, false denials, feigning amnesia and fabrication have been shown to affect memory in terms of forgetting (i.e., omissions) and false memories (i.e., commissions). Also, we show that both external and internal influences can lead to changes in the belief that an event occurred. We argue that in legal cases, triers of fact should concentrate on whether both types of influences might have affected testimonial accuracy in witnesses, victims, and suspects.

## How internal and external influences can yield similar memory effects: the role of deception and suggestion

What witnesses, victims, and suspects can accurately remember about their experiences is oftentimes a crucial issue in the court. The reason is straightforward. In many legal cases, objective evidence such as fingerprints or DNA samples is lacking (Howe and Knott, 2015). In these cases, triers of fact need to base their legal decision making on the memorial record of witnesses, victims, and suspects. Although triers of fact strive for memory reports containing a high degree of accuracy, this is frequently not what happens. Specifically, people might misremember details of, or even entire, autobiographical events. When triers of fact (e.g., judges) deem such statements to be authentic, miscarriages of justice might prevail (see Howe et al., 2018).

In the (legal) psychological literature and case reports, memory failures are often depicted as arising from external sources such as the use of suggestive interviewing techniques by the police (e.g., Otgaar et al., 2019b). However, a new branch of research has shown that memory failures can also occur because of internal influences such as lying about a crime (see for a review, Otgaar and Baker, 2018; Battista and Otgaar, 2022).

**FIGURE 1:**
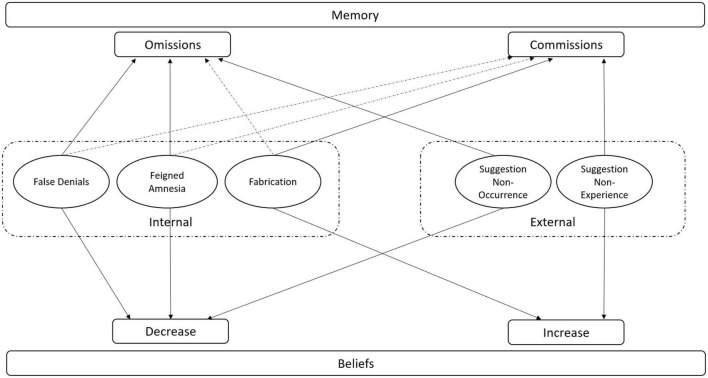
Schematic overview on how internal and external influences affect memory. In the figure, it can be seen that there are different internal and external influences and that they both can lead to changes in memory and belief. Please note that dotted lines indicate that only some studies detected a relationship between the specific factor and memory outcome.

What we will show in the current review is that these external and internal factors oftentimes exert similar effects on memory, implying that similar mechanisms might underpin these memory effects. By assembling empirical research on these themes, our review will focus specifically on two factors, namely suggestion (i.e., suggesting non-experience/non-occurrences) and lying (fabrication, feigning amnesia, false denial) as external and internal influences, respectively. The reason why we focused on these influences is that they share important similarities in how they target memories. For one thing, suggesting and fabricating non-experiences both imply the invention of details and/or events that never occurred, while suggesting non-occurrence and feigning amnesia/falsely denying details both relate to rebutting experiences that did occur (see also Figure 1). Finally, we will show how relevant and informative these findings are for legal proceedings in which memory failures might play a pivotal role determining a verdict.

## What are memory failures?

In the eyewitness memory field, much attention has been given to the following two memory failures: Omission and commission errors (e.g., Wright and Loftus, 1998; Loftus, 2005). When errors of omission take place, people who have experienced an event, fail to report (parts of) the experience. A notable example of omission errors is forgetting. Although forgetting is a normal memory phenomenon, it is often marshaled as an important memory failure (Fawcett and Hulbert, 2019). For example, forgetting is seen as a form of cognitive decline more likely to occur when getting older (e.g., Fraundorf et al., 2019). Moreover, forgetting important details of, say, a traumatic experience (e.g., sexual abuse) can be seen as a memory failure (e.g., Bell and Loftus, 1988; Ernberg et al., 2018; Shaw and Loftus, 2020) because less detailed statements can be unduly regarded as less accurate (e.g., Curci et al., 2020). However, forgetting can also be beneficial as, for instance, it aids in the facilitation of cognitive functioning (Nørby, 2015; Fawcett and Hulbert, 2019). Furthermore, a failure to report even a traumatic event (e.g., sexual abuse) does not entail an entire inability to remember it (McNally, 2005; Otgaar et al., 2019b).

Several theoretical notions have been proposed to account for the occurrence of forgetting. Classic theories of forgetting have involved principles of decay and interference (Fawcett and Hulbert, 2019). Decay theory states that forgetting takes place due to the “wasting effects of time” (McGeoch, 1932, p. 354). Most evidence, however, points to the idea that interference is the main source of forgetting (Wixted, 2004, 2005). Interference can be broadly differentiated into proactive and retroactive interference. Proactive interference refers to forgetting occurring due to prior learning affecting the retention of current information. Retroactive interference, instead, involves the negative impact of new information on previously encoded information. Research suggests that retroactive interference is the most likely candidate to explain forgetting (Wixted, 2004, 2005).

Forgetting can also be exerted intentionally (e.g., Anderson and Green, 2001; Macleod, 2012). For example, in the directed forgetting paradigm (word list variant) (Macleod, 2012), participants receive two word lists and they are instructed to forget one word list while remembering the other one. When participants are asked to recall all of the words they were presented, they typically remember fewer words from the list that had to be forgotten than the list that had to be remembered (e.g., Bjork, 1989; Bjork and Bjork, 1996; Macleod, 1999, 2012; Conway et al., 2000). Furthermore, in the Think/No Think method (Anderson and Green, 2001), participants are trained on several unrelated word pairs (e.g., ordeal-roach). Next, participants are reminded of these word pairs by being presented with a cue word (e.g., ordeal) and for each cue, one of two instructions is provided: Participants have to either recall the associated item (e.g., roach) (“think” instruction) or have to not think of the associated response (“no-think”). During the last phase, participants receive all cue words and are asked to come up with the associated words. The general finding is a memory impairment for “no-think” items compared with memory for items that were only presented during the first and last phase (Anderson and Green, 2001). This memory suppression effect has also been conceptually replicated in other studies (e.g., Joormann et al., 2005; Bergström et al., 2007).

Moreover, forgetting can also occur because of the inhibitory processes that occur in retrieval. That is, retrieval of practiced information causes the suppression of unpracticed related information (i.e., retrieval-induced forgetting effect or RIF; Anderson et al., 1994). In the RIF paradigm, participants are asked to learn a set of category item pairs (e.g., fruit–apple and drink-gin) and then are instructed to practice half of the studied pairs from half of the categories (e.g., fruit–apple). Finally, participants are asked to recall all words they can remember from the first phase. The typical finding is that the unpracticed items (e.g., pear) from the practiced categories (e.g., fruit) are more poorly recalled than unpracticed items (e.g., gin) from unpracticed categories (e.g., drink).

Although several studies have been conducted on forgetting, there are also a plethora of studies focusing on errors of commission which are instances in which people either remember events or details that were not experienced or remember them differently as compared with what they really experienced. In this category of errors there are also false memories^1^ that can be elicited spontaneously (i.e., spontaneous false memories) or because of external suggestion (i.e., suggestive false memories). Here too, false memories are commonly regarded as dangerous memory failures in the courtroom as they can lead to false accusations and miscarriages of justice (e.g., Otgaar et al., 2022a,b). To be more specific, the main contributing factor of wrongful convictions - for around 70%–is false testimony, wherein eyewitnesses have misidentified an innocent suspect during a line-up (Innocence Project, 2020). Another example concerns people who falsely remember having been abused, something that can lead to false accusations (e.g., Otgaar et al., 2019b). However, just like forgetting, the production of false memories can be seen as an integral part of a normal and adaptive memory system. Indeed, having false memories can sometimes even be beneficial in that it can aid in prospective problem-solving (Howe, 2011; Otgaar et al., 2015; Howe et al., 2017).

Theories that explain the occurrence of false memories are, for example, source monitoring framework (SMF; Johnson et al., 1993), fuzzy-trace theory (FTT; Brainerd et al., 2008), and associative-activation theory (AAT; Howe et al., 2009). According to the source monitoring framework, people make attributions about the sources of their memories (Johnson et al., 1993). When mental representations contain a high degree of memory qualities usually associated with correct recollections, people frequently attribute these mental representations to memories of experienced events. However, when these representations contain qualities such as cognitive operations (e.g., thoughts), they are more likely to be attributed to imagination or reasoning processes. False memories originate from source monitoring errors when, for example, people attribute a mental representation to a memory for an experienced event while that experience was actually suggested by someone else (e.g., a police officer; Johnson et al., 1993).

Fuzzy-trace theory stipulates that when experiencing an event, two independent memory traces are stored (Brainerd et al., 2008): Gist and verbatim traces. Verbatim traces are involved in the storage of specific details of an experience (e.g., remembering that the color of the jacket of a bank robber was red), whereas gist traces refer to the storage of the underlying meaning of an event (e.g., remembering that a bank was robbed). According to FTT, verbatim traces fade more rapidly than gist traces, making people more reliant on gist traces over time. False memories are assumed to occur when people rely on such gist traces.

Associative-activation theory uses the principle of spreading activation to explain the formation of false memories (Howe et al., 2009; Otgaar et al., 2019a). According to the tenets of AAT, when experiencing an event, this experience leads to a spread of activation through a memory network containing nodes (e.g., memories) of related experiences. This spreading activation would also activate related nodes of events that were not experienced leading to false memories. False memories are especially likely to occur when spreading activation runs rapidly and automatically through a network and when relations between nodes are strong.

In the current review, we will focus on how such memory failures can arise. Specifically, the center of our discussion lies between the impact of a specific set of external and internal influences on both forgetting (i.e., omission) and false memory production (i.e., commission). It is relevant to stress here that myriad forms of external and internal influences exist and an exhaustive review of all of these influences is beyond the scope of the current review. Here, therefore, our focus is on a certain selection of these influences, keeping the following three considerations in mind. First, our discussion will concentrate on influences in which there was an (externally or internally) “active” overt attempt to affect memory. Second, we will describe influences that are often discussed in the legal realm. Third, we discuss these influences in tandem because the available research suggests that they exert similar effects on memory. We will both discuss relevant research conducted with adult and child samples to show how these influences might affect memory.

## External influences on memory

Several studies have underlined the robust effect of suggestion on memory. Suggestion is called an external influence because oftentimes it originates from an external source (e.g., police officer, therapist, etc.). An abundance of studies using a variety of paradigms (e.g., misinformation paradigm, memory conformity, etc.) have shown that external suggestion can taint someone’s memory (e.g., Loftus, 2005). Importantly, external suggestion can take two forms. People can suggest that details/events were present while actually they were not. However, people can also falsely suggest that certain details/events were not experienced, while in fact they were. These different variants of suggestion can lead to differential effects on memory (e.g., Merckelbach et al., 2007; Af Hjelmsäter et al., 2008; Wright et al., 2009; Otgaar et al., 2010a; Frenda et al., 2011; Scoboria et al., 2017; Azad et al., 2022; Rassin, 2022). We will now describe how these different forms of suggestion can lead to very specific memory effects (see also Figure 1).

### Suggesting non-experience can lead to false memories

Several methods have been devised to investigate how suggestion of non-experienced details/events can impact memory. One of the most studied ones is the misinformation paradigm (Loftus, 2005). Basically, this paradigm follows a three-stage procedure. First, participants are presented with some stimuli (e.g., video of a burglary) or are involved in an interactive event (e.g., a science demonstration). Then, participants receive misinformation in the form of, for example, an eyewitness account containing false details (e.g., that the burglar stole jewellery while money was really stolen). Finally, a memory test is provided in which participants have to state which details they can still recollect. The misinformation effect refers to the finding that suggested false details are often reported by participants as having occurred during the first phase (Frenda et al., 2011).

Another paradigm used to externally engender entire false autobiographical experiences is the false memory implantation paradigm (Loftus and Pickrell, 1995). In this paradigm, participants are asked to report what they can still remember about events that ostensibly happened to them in their childhood. The important manipulation is that one of the events is false (i.e., being lost in a mall), being fabricated by the experimenters. After multiple interviews, during which the researchers suggested participants had experienced the false event, the canonical finding is that about 30% of participants fall prey to the suggestion and report having experienced such false event (Scoboria et al., 2017). Researchers have successfully implanted a wide array of false autobiographical events that share characteristics with events such as sexual abuse. For example, researchers have succeeded to implant painful events (e.g., being bitten by a dog; Porter et al., 1999), shameful events (e.g., swimming trousers falling off during swimming; Otgaar et al., 2021), and events that allegedly occurred more than once (Calado et al., 2021).

An additional way to evoke false memories is the memory conformity paradigm (Wright et al., 2009). This paradigm has been used with three variants. In the first one, pairs of participants are presented with stimuli (e.g., picture of a desk). Participants are under the impression that they are witnessing the same stimuli, but each participant is viewing a slightly different version of the stimuli. For example, one participant might see a pen on the desk, while the other participant is presented with a desk without a pen. After the encoding phase, participants have to recall the stimuli collaboratively. What happens here is that participants will (unintentionally) suggestively influence each other’s statements. During a final memory test, participants have to individually report what they can still remember concerning the stimuli.

In the second variant, group of participants are presented with the stimuli and, then, they discuss such stimuli. However, some participants are confederates of the experimenter who provide misleading information as actual elements of the stimuli. Finally, in the last variant, participants are simply presented with information of what was said by co-participants. Overall, these studies found that participants report having seen details that were actually suggested/discussed by the other participants (Wright et al., 2009).

Collectively, these paradigms largely show that external suggestion can lead to the production of false memories. Several theoretical explanations exist to explain how suggestion can foment false memories creation, and one of the most popular ones is the source monitoring framework, such that false memories due to external suggestion are basically source monitoring errors (e.g., Lindsay and Johnson, 2000).

### Suggesting non-occurrence can lead to omission errors

Apart from suggesting that an event or detail was experienced while it was not, the reverse can also take place. That is, one can suggest that something was *not* experienced, while it actually was. Studies using this variant of suggestion have shown that this can lead to omission errors or failures to report experienced events (e.g., Pezdek and Roe, 1995; Wright et al., 2001; Merckelbach et al., 2007; Af Hjelmsäter et al., 2008; Otgaar et al., 2010a; Azad et al., 2022).

Compared with work on suggesting non-experiences, empirical research focusing on the suggestion of non-occurrence is quite limited. To study this, researchers have simply tweaked the usual false memory methods and focused on suggesting non-occurrences instead of suggesting non-experiences. For example, in one of the first of these studies focusing on suggesting non-occurrences conducted by Pezdek and Roe (1995), 4- and 10-year old children were touched in a specific way (e.g., hand on the children’s shoulder) or not touched at all. Children were told that a different touch, a new touch, or no touch at all had happened. Of relevance to the current discussion is the condition in which children were touched but were told that nothing occurred. The authors found that children were not likely to accept the suggestion that no touch occurred and, hence, did not demonstrate significant more omission errors in their memory reports than children who did not receive the suggestion that no touch occurred.

Other studies, however, have shown that suggestion of non-occurrences *can* lead to omission errors. In two experiments, Wright et al. (2001) showed that post-event information suggesting that event was not experienced could make the memory concerning that event less accessible. In their experiments, participants saw certain stimuli (e.g., a restaurant scene depicted in slides). After this, they were again provided with these stimuli, but a critical scene (e.g., waitress taking an order) was omitted. Participants were instructed to use these stimuli to generate a story (Experiment 1) or imagine a scene (Experiment 2). During a final memory task, the important result was that the post-event omission led people not to report the critical scene in free recall and recognition. This effect has also been demonstrated when children were involved as participants (Williams et al., 2002).

In addition, recent work has extended our prior understanding of the memory consequences of suggestions of non-occurrence by further investigating this issue in a sample of adults (Azad et al., 2022). In three studies, Azad et al. (2022) asked participants to watch a video (i.e., child kidnapping case) and then exposed them to suggestions of non-occurrence once (Studies 1 and 3) or multiple times (Studies 2 and 3). In a final stage, participants’ memory for the video was tested. Interestingly, single suggestions of non-occurrence did not make participants prone to omissions, but they did find that repeated suggestions of non-occurrence led participants to omit video-related information.

The just-mentioned studies used a rather subtle manipulation to induce omission errors. In Otgaar et al.’s (2010a) study, younger (4–5-year-olds) and older children (9–10-year-olds) had to remove three pieces of clothing of a puppet. In one condition, it was suggested to the children that they actually removed two pieces of clothing. This was done using a verbal suggestion and false evidence (putting one piece on the puppet again without the child noticing it). The authors found that although children initially claimed to take off three pieces of clothing, after the suggestion, a significant minority of children reported to have only removed two pieces of clothing (Otgaar et al., 2010a; see for similar results, Merckelbach et al., 2007; Af Hjelmsäter et al., 2008). Moreover, in a second study, Otgaar et al. (2010b) asked children to erroneously report that they only removed two pieces of clothing. This group had to complete a choice reaction time task consisting of pictures of different types of clothing. Their instruction was to indicate whether they removed these pieces of clothing or not. The primary result was that children made significantly more errors for removed pieces of clothing that they failed to report than for those they had not removed.

Contrary to the formation of false memories, little attention has been paid to the mechanisms underpinning omission errors. Pezdek and Roe (1995) referred to terms such as “erasing” memories thereby implying that the suggested memory is gone or –to use a less dramatic connotation– has become inaccessible to retrieval processes. However, it has also been shown that at least for omission errors in children, erasure was not a viable candidate to explain the failure to report experienced events (Otgaar et al., 2010b). Thus, a more promising explanation for omission errors is that suggesting non-occurrences does not impact the recollection of experienced events, but the belief that a particular event occurred. Alternatively, omission errors might simply refer to failures to report remembered information.

### Suggestion (of non-experience and non-occurrence) can lead to false beliefs and non-believed memories

Previous research has mainly focused on the impact of suggesting non-experiences and non-occurrences on false memories and omission errors. However, recent research shows that these suggestions can even have more subtle effects on true and false memories. A recent surge of experimentation has shown that suggestion can impact the belief that an event occurred rather than the recollection of an event (e.g., Mazzoni et al., 2010; Clark et al., 2012; Otgaar et al., 2014b; Li et al., 2020). Believing that an event took place and recollecting an event are two different concepts contributing to the phenomenology of remembering. Belief refers to trusting that an event occurred, while recollection refers to re-experiencing an event including vivid images concerning this event (Scoboria et al., 2004, 2014). For perhaps most of our memories, we are prone to believe that an event occurred *and* to have vivid recollections concerning that event. However, for certain events, believing and recollecting are detached from each other. For example, people believe that they were born, but have no recollection of that event. Interestingly, for certain experiences, people have vivid recollections of an event, but no longer believe in the occurrence of that particular event. This latter type of memory has been called non-believed memories (Mazzoni et al., 2010; see for a review, Otgaar et al., 2014b). This counterintuitive memory phenomenon has stirred an abundance of research as it might clarify how suggestion can shape memory.

For example, it has been suggested that before a false memory for an event can be evoked, the event should be first considered plausible and then a belief that the event has occurred should have been formed (e.g., Mazzoni et al., 2001). This implies that suggesting non-experiences might also lead to false beliefs. This is indeed what research has been showing. For example, Scoboria et al. (2012)–using the false memory implantation paradigm–found that false suggestions increased false beliefs of a non-experienced event. Furthermore, Scoboria et al. (2017) performed a mega-analysis on false memory implantation studies and it was found that participants often expressed high belief in the occurrence of the falsely suggested event.

However, the reverse can occur as well. That is, when suggestion is provided about non-occurrences, belief can be affected as well. An increasing body of research is showing that suggesting non-occurrences can lead to reductions in belief and even end up in non-believed memories. In two experiments, Otgaar et al. (2013) used the false memory implantation paradigm to induce false memories of a hot air balloon ride. Adults (Experiment 1) and children (Experiment 2) were suggestively told that they experienced a hot air balloon ride when they were younger during multiple suggestive interviews. Importantly, when they were debriefed about true nature of the study, they were asked whether they still believed in the occurrence of the false event and still had recollections concerning the false event. The principal finding was that a significant proportion of subjects (13% for adults and 15% for children) developed non-believed memories after debriefing. That is, they still had a memory of going in a hot air balloon, but no longer believed that the event happened. Follow-up studies have confirmed the suggestion that non-occurrences can lead to belief changes and result in non-believed memories when (1) different paradigms are used (e.g., Clark et al., 2012), (2) suggestion is provided on true and false memories (e.g., Scoboria et al., 2012; Mazzoni et al., 2014), and (3) children and adults are tested (e.g., Otgaar et al., 2017). In addition, more recently, in two studies, Li et al. (2020) tested whether non-believed memories can also be reported for bizarre events in the standard imagination inflation paradigm. They asked participants to perform or imagine both simple familiar actions and bizarre actions. After 1 day, participants were invited to imagine simple actions of which some were new actions and some were actions performed the day before. After a week, participants completed a memory task and, when some actions were (correctly or incorrectly) recognized as performed, they were negatively challenged (i.e., participants were told that the action was not performed). The authors found that challenging actions that participants claimed to have performed decreased beliefs in these actions and led to the production of non-believed memories both for bizarre actions and familiar actions.

To recap, external influences can affect memory in different forms. When someone is suggestively told that an event or detail was experienced, while in fact this was not, a multitude of studies shows that such suggestions can facilitate the formation of false beliefs and false memories. Furthermore, when someone is suggestively told that a certain event did not occur while in fact it did, research indicates that it can lead to omission errors, belief reductions, and even non-believed memories. We now turn our attention to influences that are exerted internally and how they might contaminate memory performance.

## Internal influences on memory

An increasing body of research is currently showing that deception is a powerful internal influence that can affect memory (e.g., Otgaar and Baker, 2018; Paige et al., 2022; Vo et al., 2022). Vrij (2008) defined lying as a “a successful or unsuccessful deliberate attempt, without forewarning, to create in another a belief which the communicator considers to be untrue” (p. 5). According to this definition, lying is exerted intentionally *by the one* exercising the lie. This is relevant because self-generation might lead to stronger memory contamination because it could be speculated that such self-generation makes the lie also more personally relevant (e.g., Howe et al., 2013; Wang et al., 2019; but see also Pezdek et al., 2009). Even though lying occurs on an almost daily basis in everyday life (Riesthuis et al., 2022a), this behavior is legally relevant because it is often exerted by suspects, victims, and witnesses (e.g., Vrij, 2008; Otgaar and Baker, 2018; Verigin et al., 2019).

Several deceptive strategies can be exercised and evidence is accruing that different forms of deception can lead to different memory effects. Otgaar and Baker (2018) argued that these differential memory effects might be caused by differences in cognitive resources that are needed to exercise certain types of lies (see also Battista et al., 2021a,c). In this section, we will focus on the memory effects of three types of lying: Fabrication, false denials, and feigning amnesia (see also Figure 1).

### Fabrication can lead to false memories (and forgetting)

In legal contexts, fabrication is a common phenomenon. For example, perpetrators might willingly distort the truth or invent an entire story in order to mislead the police. Still witnesses and victims might come up with false information while answering police interviews, such as accusatorial interviews (Garven et al., 1998; Meissner et al., 2014). One of the most often-used methods to examine the impact of (self-generated information) fabrications on memory is the forced confabulation paradigm (Ackil and Zaragoza, 1998). In the first study using this paradigm, children and adults viewed a clip from a movie. After viewing the movie, participants were instructed to answer some questions concerning the movie. In the forced confabulation group, participants were told to provide an answer to every question and guess if they did not know the answer. By contrast, participants in the control condition only had to answer questions of which they were sure they knew the answer and were instructed not to guess. Importantly, participants were presented with questions about details that were presented in the movie, but were also presented with questions about non-presented details (e.g., a question about what was stolen when actually nothing was stolen). One week later, all participants received a source memory task. Specifically, they were asked whether they talked about certain details the week before and whether they saw these details in the video. The most important finding was that participants who were in the forced confabulation group claimed to have seen their own confabulations in the movie. In other words, forced confabulations led to the production of false memories for the confabulated responses.

Subsequent research has extended this work and, for example, showed that forcing participants to fabricate entire events (instead of details) can also generate false memories (e.g., Pickel, 2004; Chroback and Zaragoza, 2008). For example, Chroback and Zaragoza (2008) had participants view a clip from a movie (i.e., Looking for Miracles). Two days later, participants had to answer several interview questions of which some referred to false events. Participants were explicitly instructed to provide an answer to every question and guess if they did not know the answer. One week and 8 weeks after viewing the movie clip, participants received a recognition and recall test, respectively. Although false memory formation was limited after 1 week, after 8 weeks, participants claimed to have seen their own forced confabulations nearly 50% of the time.

Furthermore, apart from using the forced confabulation paradigm, other related research has also shown that self-generated fabrications can lead to false memories. In fact, Pickel (2004) showed that participants who fabricated misinformation themselves started to falsely remember this misinformation as being true. Schreiber et al. (2001) showed that when children were instructed to speculate about what objects could do, after a 5–6-month delay, children formed false answers to what these objects could do. Specifically, in their study, children received atypical actions for common objects (e.g., throwing a knife away). One week later, children were asked to speculate what else these objects could do (e.g., “What else can could he have done with a knife?”). The researchers found that inviting children to speculate could lead to false answers of these speculations at follow-up memory tests.

In short, lying, in the form of fabrication, can lead to the formation of false memories and this effect seems also to be not mitigated or exacerbated by other factors (e.g., incentive to lie, cognitive resources, personality traits) as shown in some recent experiments (Battista et al., 2021b,c, 2023; Riesthuis et al., 2022b; Battista et al., under review^2^). All these studies suggest that one explanation for this effect is that, just as false memories, fabrications can result in source monitoring errors because the fabrications appear phenomenologically similar to memories of experienced events.

Meanwhile, there is some limited evidence demonstrating that fabrication can also engender forgetting effects as well. For example, Pickel (2004) not only showed that self-generated misinformation was misremembered but that it also led participants to remember less about the target stimulus. A similar finding was observed by Riesthuis et al. (2022b) who found that creating a false alibi not only generated false memories but also resulted in omission errors. A possible interpretation for why fabrication led to forgetting is because the act of fabrication prevented participants to rehearse the experienced stimuli. This lack of rehearsal might have led to the forgetting of details concerning the event (see also Pickel, 2004; Riesthuis et al., 2022b).

### False denials can lead to forgetting and false memories

A simpler deceptive strategy than fabrication is falsely denying that an experienced event unfolded. There is a vast literature showing that offenders of violent crimes (e.g., homicide, sexual abuse) oftentimes falsely deny that they committed a criminal act (e.g., Henning et al., 2005; Watson et al., 2016). Furthermore, false denials have been mentioned as one of several strategies that victims use to cope with sexually abusive experiences (Romeo et al., 2018; Ahern et al., 2019; Bücken et al., 2022c). A fundamental question here is whether the act of false denials might have memory impairing effects.

Research is amassing revealing that false denials can lead to omission errors. In the first study on this issue, Vieira and Lane (2013) instructed participants to study pictures of different objects (e.g., teacup). After this, participants received studied and unstudied objects and had to tell the truth or deny seeing these objects. The consequence was that for certain objects, they falsely denied studying these objects. Following this, participants were presented with a source memory test. Of relevance for the current discussion was the finding that participants forgot having falsely denied certain objects.

Otgaar et al. (2014a) found similar memory effects of false denials. In their experiment, they adapted the forced confabulation paradigm and added a false denial condition. Specifically, in their experiments, children (6–8- and 10–12-year-olds) and adults viewed a video and then received a memory test about details of that video. After this, participants were invited to lie (i.e., falsely deny or fabricate) or tell the truth about what seen in the video. Of importance for the current discussion are the false denial and control conditions. After the memory test, participants in the false denial condition had to falsely deny seeing certain details while control participants had to tell the truth. One week later, participants received a source memory test in which they were asked whether they talked about certain details and whether they saw certain details in the video. The most interesting finding was that participants in the false denial forgot they had talked about certain details which in fact they did. This memory impairing effect of false denials has been dubbed denial-induced forgetting (Otgaar et al., 2016b).

After this first demonstration, the denial-induced forgetting effect has been observed using various stimuli like pictures (Otgaar et al., 2016b), virtual reality (Romeo et al., 2019) or daily life actions (Li et al., 2022a,b), and memory tasks (i.e., recognition and recall) (Otgaar et al., 2018). Taken together, false denials have been shown to lead to omission errors and especially omissions errors for details that were discussed rather than to the forgetting of the event.

Although some studies have found that false denials - in specific circumstances–might undermine our memory for the event, this work is limited. For instance, Battista et al. (2020) asked participants to repeatedly deny certain details while denying other details only once. They demonstrated that when details were denied four times, correct recall levels were lower than when details were denied once. Still, a detrimental effect of false denials on memory for the original event was also found by Romeo et al. (2019) in a study in which they tested the mnemonic impact of falsely denying emotional events. Similarly, another recent study (Battista et al., 2021a) found that when the false denials strategy requires a high involvement of cognitive resources to be employed, it can also result in a forgetting effect for the event (but see also Li and Liu, 2021).

Recent experimentation has shifted attention to the question whether false denials might also affect false memory production. The reasoning here was as follows. If false denials lead to omission errors then, based on theories such as FTT and AAT, such omission errors should affect the risk of false memory production. AAT would, for example, predict that when omission errors occur, activation will spread less to neighboring nodes thereby reducing the production of false memories (Howe et al., 2009). Evidence for this was found by Otgaar et al. (2020). They showed participants lists containing associatively-related words (e.g., tears, sorrow, grief) linked to a non-presented theme word (i.e., cry). After the encoding phase, half of the participants had to falsely deny seeing these words, while the other half had to tell the truth. During a final memory task, participants who had to deny created fewer false memories than truth-tellers (Experiment 1).

Recently, Bücken et al. (2022a,b), however, demonstrated that false denials can increase people’s willingness to go along with false information. In one of their studies (Bücken et al., 2022b), participants viewed a video of a car crash and following this, half of them falsely denied that certain details were in the video while others had to tell the truth. After 1-week participants received misinformation concerning what happened during the interview and the car crash. False denials increased susceptibility to misinformation concerning the interview.

Scholars suggested that a lack of rehearsal might be a possible mechanism to explain the mnemonic consequences of false denials (Otgaar and Baker, 2018). Nevertheless, there are some recent indications that inhibition could be the mechanism underpinning the denial-induced forgetting effect and, occasionally, a forgetting of the event (Otgaar et al., 2020). The rationale here is that during the act of denial, retrieval of the-to-remembered event is temporarily inhibited leading to forgetting effects.

### Feigning amnesia can lead to forgetting and false memories

A deceptive strategy that is also oftentimes used by offenders of violent crimes is pretending to suffer from memory loss for such event (e.g., Cima et al., 2002; Pyszora et al., 2003; Jelicic, 2018). Offenders claim amnesia for several reasons such as obstructing police investigations and interfering with legal proceedings (Tysse, 2005; Tysse and Hafemeister, 2006). In general, prevalence data show that about 30% of offenders who have committed violent crimes claim memory loss (see for a review, Mangiulli et al., 2021). Like other deceptive strategies such as fabrication and false denials, feigning amnesia has been shown to exert memory undermining effects.

Specifically, an increasing corpus of studies have shown that feigning amnesia can foster omission errors (e.g., Christianson and Bylin, 1999; Bylin and Christianson, 2002; Van Oorsouw and Merckelbach, 2004; Sun et al., 2009; Mangiulli et al., 2018a,b, 2019a,2019b). In the first study of this kind (Christianson and Bylin, 1999), participants were presented with a description of a crime and had to imagine being the offender of that particular crime. During a memory test, one group was instructed to feign memory loss for the crime while another group had to report the same event truthfully. One week later, all participants had to respond truthfully during a final memory test. The central finding was that those participants who feigned amnesia remembered fewer details (i.e., omissions) than truthful responders. Since then, research has replicated this effect using different stimuli (e.g., Van Oorsouw and Merckelbach, 2004; Sun et al., 2009; Mangiulli et al., 2018a,b). The memory undermining effect of feigning amnesia has been mostly attributed to a lack of rehearsal (but see for an alternative explanation, Mangiulli et al., 2019b). Indeed, studies that included a third group that was only tested after a delay showed that those feigning amnesia and participants in this delayed control did not statistically differ from each other in terms of memory performance. The reason for this is because both groups were less likely to rehearse the stimuli than the honest control group (Bylin and Christianson, 2002; Van Oorsouw and Merckelbach, 2004).

Apart from the finding that feigning amnesia can lead to omission errors, studies have also revealed that it can engender false memory creation. The explanation behind this is that feigning amnesia does not involve a single concrete deceptive strategy (see Mangiulli et al., 2020, 2021). That is, people who choose to feign amnesia can do so by just claiming memory loss (“*I do not remember*”), but also by adding fictitious details to their amnesic claim (“*I cannot remember because I was somewhere else during the crime*”). Interestingly, research in which participants were specifically instructed not to just deny the experience (and thus potentially fabricate details) demonstrated that they had elevated levels of false memories (Van Oorsouw and Giesbrecht, 2008). Recent research has shown that those feigning amnesia who decided to omit information were the ones with the lowest memory performance while those who also added false details in their feigned account for a crime reported the highest amount of commission errors (Mangiulli et al., 2019b,^2020^).

However, when Mangiulli et al. (2020) examined whether feigning amnesia would also increase the risk of reporting misinformation, no evidence was observed. Thus, although the act of simulating amnesia can lead to errors of omission and commission, it does not seem to increase people’s susceptibility to external pressure.

### Lying can lead to changes in belief

Lying not only leads to false memories and omission errors, but there is some research showing that it can also affect the belief that an event took place (Polage, 2004, 2012, 2019; Romeo et al., 2018). For example, Polage (2004) asked participants to rate the likelihood that certain events happened to them before the age of ten. Approximately 2 weeks later, participants had to falsely claim to an experimenter that they experienced an event that they previously rated as unlikely to have happened them. One week later, participants had to truthfully rate the same events and indicate how likely it was that these events happened to them before the age of ten. In general, participants rated the events as less likely to have occurred to them after lying about them: An effect called fabrication deflation. However, what was also found was that a small subset of participants (10–16% in two studies) were more likely to claim that the events happened to them after lying about them, which Polage (2012) referred to as fabrication inflation effect. Interestingly, individual differences might play a role in this fabrication inflation effect as there is some preliminary evidence showing that high levels of dissociative experience might be positively related to the fabrication inflation effect (Polage, 2012).

So, it seems that fabrication might lead to increases in the belief that a non-experienced event occurred (but see also Riesthuis et al., 2020). Recent research has also focused on whether false denials might lead to decreases in the belief that an event occurred. Otgaar et al. (2016b) examined the denial-induced forgetting effect and compared a group that had to falsely deny that certain details were experienced and a group that was falsely suggested by an experimenter that certain details were not experienced. Decreases in belief were not found for the false denial group. However, Polage (2019) used a similar methodology in her fabrication inflation work and included a false denial condition and she did find that false denials led to decreases in the belief that events were experienced. Furthermore, Romeo et al. (2018) showed that feigning amnesia led to decreases in belief as well as recently Li et al. (2022b) found that when people mix different type of lies (i.e., false denial and fabrication) their beliefs for the occurrence of event-related details can decrease.

Taken together, the work on internal influences and memory, and more specifically the work on the impact of lying on memory, has shown that lying has differential effects on memory. Fabrication has been shown to lead to increases in belief in the occurrence of the self-generated information and false memories, while false denials have primarily been found to lead to omission errors. In addition, depending on which specific strategy is used, feigning amnesia has been found to lead to belief changes, omission errors, and false memories (see also Figure 1).

## Future perspectives

Despite one being other-generated and the other self-generated, it is evident throughout this review that external and internal influences oftentimes exert similar effects on memory. For example, as displayed above, suggesting false experiences as well as self-generating false information (and feigning amnesia) can lead to false memories. Moreover, suggesting non-occurrence and falsely denying or feigning amnesia for experienced event can both lead to forgetting. Finally, it appears that both types of influences can impact the belief in the truth value of an experienced event.

Having established the effects of both external and internal influences on memory, a timely question arises. This question–which likely could orient future research paths–concerns examining whether these effects are (partially) driven by a common mechanism. We propose that one such a mechanism could be cognitive dissonance (i.e., a displeasing psychological state caused by a mismatch between two or more elements in a cognitive structure; Festinger, 1957, 1962). That is, dissonance is thought to play an essential role in whether belief is reduced or not when suggestion is provided about non-occurrences (Scoboria et al., 2014). According to the model postulated by Scoboria and Henkel (2020), when someone is suggestively told that their memory is incorrect, dissonance arises. Such dissonance can be at an interpersonal or intrapersonal level. Concerning interpersonal dissonance, the idea is that people would evaluate the costs and benefits of (dis)agreeing with the suggestion. If people agree with the suggestion, a reduction in belief might take place. On an intrapersonal level, instead, people would evaluate the suggestion with their own memory (e.g., if the suggestion pertains to a memory which is vague). Here too, if the suggestion is accepted, it is likely that belief in the occurrence of an event will be undermined. Evidence of this possibility comes also from recent studies investigating a possible relationship between non-believed memories and memory distrust (i.e., people’s distrust toward their own memories) (Zhang et al., 2022a,b). These studies found support for a positive association between memory distrust and non-believed memories such that memory distrust was assumed to be a reason why people reduce beliefs in the occurrence of events. In addition, the idea that cognitive dissonance can play a crucial role in how internal and external influences can affect our memory comes from work–albeit limited–showing that dissonance is also related to the production of false memories and lying (e.g., Merckelbach and Merten, 2012; Rodriguez and Strange, 2015). For example, Rodriguez and Strange (2014) had participants make an easy or difficult choice between two smartphones. Following this, participants were instructed to remember their decision experience. Participants receiving the difficult choice experienced cognitive dissonance and were more likely to misremember their initial decisions than participants receiving the easy choice.

Cognitive dissonance might also be related to when people lie and then come forward with the truth. That is, some lies (e.g., false denials) might be displeasing if they are exercised under pressure and hence, create a mismatch with a memory for an experienced event. Therefore, based on the proposition that cognitive dissonance is assumed to play a role in in how external and internal influences affect memory, several specific future research enterprises and predictions can be postulated. For example, if dissonance plays a role in how false denials lead to omission errors, then the following might be expected: Omission errors would be more likely to occur when, for example, the memory of the experienced event is weak because of high intrapersonal dissonance. The reason is because when dissonance takes place, people will simply try to resolve it. So, people would only agree to the false denial of the event if the denial does not conflict too much with their own experience. This means that when people have difficulties in remembering an event, the act of false denials will more likely be accepted, hence leading to omission errors. A possible way to empirically test this idea in future studies is by having participants experience an event and then assigning some of them to a group that has to immediately deny experiencing the event, while others have to falsely deny experiencing the event a week later (i.e., delayed group). The prediction would be that the latter group will have a weaker memory performance for the event than the other one, making it more susceptible to intrapersonal dissonance. This, in turn, would lead the false denials to robust memory undermining effects.

Beyond the idea of testing a possible effect of dissonance, there are also other routes that might be fruitful to explore. For example, one interesting avenue is to examine when external or internal influences affect memory and/or belief. Based on earlier models and research (e.g., Mazzoni et al., 2001; Scoboria et al., 2004, 2014), the idea is that people first form a belief that an event happened and after that a recollection of an event is created. This work has also shown that beliefs are more malleable than recollections (e.g., Otgaar et al., 2014b). A critical question for future experimentations is to investigate whether manipulating the levels of the impact of external/internal influences can divergently affect beliefs and recollections. That is, there might be some forms of dose-response relationship in that weaker forms of external/internal influences (e.g., subtle external suggestion using misinformation in an eyewitness testimony) are more likely to affect belief, while stronger forms of external/internal influences (e.g., a policeman providing harsh suggestive interviewing tactics) are more likely to target recollection.

One might also wonder whether the observed effects of lying on memory are perhaps due to fact that participants were “instructed” to lie, while in real life settings, witnesses, victims, and suspects frequently choose to lie. An imperative question is to empirically test the proposition that “instructed” lies have different effects on memory than “voluntary” lies. Although limited, recent research has examined whether the volitional act of lying has different effects on people’s memory than when they are instructed to lie. Interestingly, these studies observed that similar memory undermining effects are detected when participants can freely choose to deceive, thereby suggesting that the act of lie is the determining factor in the observed memory effects (Dianiska and Meissner, 2022; Li et al., 2022a; Riesthuis et al., 2022d). Of course, future research could increase the knowledge base in this area and attempt to replicate these recent studies.

Another important avenue for research could be investigating what are the memory consequences caused by the interplay of different influences. Indeed, in the current review, we have focused on how lying and external suggestions can taint memory and result in forgetting and false memories. However, it is important to be cognizant of the fact that such memory failures can also arise because these influences might well work in tandem (e.g., Mangiulli et al., 2020; Bücken et al., 2022a,b; see text footnote 2). The investigation of such interactions might result into a more all-encompassing understanding on how different types of influences impact memory.

## Legal implications

Wrongful convictions can be caused by memory failures. For example, suggestive therapeutic sessions can lead to false memories of sexual abuse leading to false accusations and miscarriages of justice (Howe et al., 2018; Otgaar et al., 2019b). Also, data from the American Innocence Project has revealed that about 70% of wrongful convictions were the result of eyewitness misidentification (Innocence Project, 2020; see also Wells and Olson, 2003; Saks and Koehler, 2005). Such misidentifications, which may lead innocent people to be imprisoned, are memory failures and they have received a wealth of empirical attention within the psychological and legal realm. Importantly, such false positives are sometimes regarded as more serious than false negatives (acquitting a guilty person), an adage also known as the Blackstone ratio (Blackstone, 1765; see also de Keijser et al., 2014).

However, likely because of sentiments as the Blackstone ratio, other memory impairments (e.g., forgetting) perhaps did not receive so much attention as the former. However, omission errors and not believing that a certain event took place can have egregious consequences in police investigations and legal cases. For example, when witnesses are unable to remember how a certain criminal experience exactly unfolded, it might become difficult for the police to find a suspect, wasting unnecessary resources. Also, if a victim of abuse expresses low belief that the abusive event truly happened, an accusation might not be taken seriously and would hinder fact-finding in a criminal investigation. But also suggesting non-experiences to victims, witnesses, and suspects might lead to misidentifications and false confessions (e.g., Zajac and Henderson, 2009; Frenda et al., 2011; Scherr et al., 2020), such as when the perpetrators silence their victims claiming that nothing happened (e.g., Shepp et al., 2019).

Interestingly, we have additionally shown that memory failures such as forgetting and false memories can not only be prompted by external influences, but can also be initiated by means of internal influences. What we have shown is that false denials (and feigning amnesia too) can result into forgetting and decreases in belief, while fabrication (and as well as feigning amnesia in certain circumstances) can boost false beliefs and false memory formation. Collectively, this work has demonstrated that lying can exert similar effects on memory as external influences. However, from a practical perspective, research on how lying affects memory is still limited (see also Battista and Otgaar, 2022). The issue of lying has often been examined, but this examination is predominantly in the context of deception detection (e.g., Granhag et al., 2015). Although research in the area of deception detection sometimes uses principles of memory (e.g., recognition) to detect concealed knowledge (e.g., Verschuere et al., 2011), the work described here ascribes causal effects of lying on memory, clearly demonstrating that the act of lying can have deteriorating effects on memory. This pattern of results can be relevant for different professionals working in the legal arena. For example, memory researchers working as expert witnesses are often asked to estimate the reliability of testimonies of witnesses, victims, and suspects (Otgaar et al., 2017). What such expert witnesses basically do is to evaluate whether statements might have been affected by, for example, suggestive interviewing techniques. However, so far, the impact of internal influences on testimonial accuracy is not clear yet. To give a case example, it is common that victims of sexual abuse falsely deny being abused and only after a certain period of time come forward with the truth (Magnusson et al., 2017). Memory experts who are asked to evaluate the reliability of this victim’s statement might now also note that false denials can have detrimental effects on memory too. Similarly, legal professionals (e.g., police officers), who know that fabrication can result into false memories, would dismiss the use of coercive and accusatorial interrogations that make the interviewee more likely to come up with false information for the forgotten crime-related details (Garven et al., 1998).

On a related note, one issue that has to be at the foreground concerning the legal implications of the reviewed work is to what extent effects observed in experiments on external and internal influences on memory are meaningful and practically relevant. That is, although the reviewed literature shows that these influences can negatively impact memory, a basic but forthright question is whether these findings have any practical meaning. So, experiments conducted in this area should establish certain effect sizes and the question is whether such effect sizes bear any relevance in actual legal cases. This question can only be answered if, as a field, we agree to some extent on which effect sizes are of relevance in practical settings. In other words, the problem is understanding to what extent the achieved results of psychological studies are sufficiently informative and can bring a significant contribution for legal professionals’ practice (Riesthuis et al., 2022c). Specifically, this question is related to what the smallest effect size is of interest in these memory experiments (see Lakens et al., 2018). That is, we recently argued that memory experiments should contain elements that can generate effects of interest for the (legal) field (see Otgaar et al., 2022b). If we consider what the smallest effect size is of interest for research on external and internal influences on memory, our argument is that even when such influences (e.g., false denial, suggestion non-experiences) lead to increases or decreases of only one (falsely) remembered detail, this might be of high value to the legal field. This is vital because even one remembered (or forgotten) detail can be determining, for instance, in the reconstruction of the crime (see also Mangiulli et al., 2021; Riesthuis et al., 2022b). Taken together, establishing which effects are of interest concerning the impact of external and internal influences on memory might lead to stronger experiments to demonstrate such effects. This might also reveal whether internal or external influences evince larger effects on memory and which one might be more practically relevant. Hence, we believe that if memory researchers are planning new studies and want to conduct an *a priori* power analysis, they should estimate which effect size is needed to establish an effect of interest. If the field agrees that effect sizes such as the one explained above (i.e., increase/decrease one remembered/forgotten detail) are of practical interest, stronger memory experiments can be built.

## Concluding remarks

In what way can memory be shaped? In the present review, we have demonstrated that external and internal influences can exert similar effects on memory. Specifically, we showed that forgetting and false memories can arise when people are exposed to suggestion of non-occurrences and non-experiences, respectively. Similarly, such forgetting and false memories can also be produced when people falsely deny, feign amnesia for, and fabricate events. Furthermore, we have demonstrated that these influences can also lead to amplifications and reductions in the belief that an event occurred. We speculated that focusing on whether cognitive dissonance might be a centerpiece mechanism will likely engender novel research on how internal and external influences can shape memory.

## Author contributions

HO conceived the project and wrote the manuscript. MH, IM, and FB wrote the manuscript and critically revised it. All authors contributed to the article and approved the submitted version.

## Footnotes

1 In the current paper, we use the term “false memories” as referring to remembering non-experienced details/events and referring to instances in which details of events are remembered differently (also called memory distortions). Also, we use the terms omissions and forgetting, and commissions and false memories interchangeably in the current manuscript.

2 Battista F., Otgaar H., Riesthuis P., and Mangiulli, I. (under review). *Lying on misleading information: False confirmation leads to fabricated memories*.
